# Inhibition of Nutlin-Resistant HDM2 Mutants by Stapled Peptides

**DOI:** 10.1371/journal.pone.0081068

**Published:** 2013-11-20

**Authors:** Siau Jia Wei, Thomas Joseph, Sharon Chee, Ling Li, Larisa Yurlova, Kourosh Zolghadr, Christopher Brown, David Lane, Chandra Verma, Farid Ghadessy

**Affiliations:** 1 p53Lab, Agency for Science Technology and Research, Singapore, Singapore; 2 Bioinformatics Institute, Agency for Science Technology and Research, Singapore, Singapore; 3 ChromoTek GmbH, Planegg-Martinsried, Germany; 4 School of Biological Sciences, Nanyang Technological University, Singapore, Singapore; 5 Department of Biological Sciences, National University of Singapore, Singapore, Singapore; University of Hawaii Cancer Center, United States of America

## Abstract

Pharmacological modulation of p53 activity is an attractive therapeutic strategy in cancers with wild-type p53. Presently in clinical trials, the small molecule Nutlin-3A competitively binds to HDM2, a key negative regulator of p53 and blocks its activity. We have described resistance mutations in HDM2 that selectively reduce affinity for Nutlin but not p53. In the present communication, we show that stapled peptides targeting the same region of HDM2 as Nutlin are refractory to these mutations, and display reduced discrimination between the wild-type and mutant HDM2s with regards to functional abrogation of interaction with p53. The larger interaction footprint afforded by stapled peptides suggests that this class of ligands may prove comparatively more resilient to acquired resistance in a clinical setting.

## Introduction

The p53 tumor suppressor plays a key role in governing cell fate [1,2]. Aberrations in p53 function, typically arising through point mutation are seen in 50% of all cancers [3,4]. Hence significant effort has been made towards the pharmacological restoration of wild-type function in mutant p53 [5-7]. In malignancies with wild-type p53 status, tumour-specific up-regulation of p53 activity is a therapeutic strategy actively being explored [8]. To this end, an array of inhibitors designed to block interaction of p53 with its key negative regulator, HDM2, have been developed [9-11]. p53 is the substrate for the ubiquitin ligase activity of HDM2 which targets p53 for proteosomal degradation [12-15]. The small molecule Nutlin-3a (hereafter termed Nutlin) competes with p53 for binding to an extended hydrophobic groove in the N-terminal domain of HDM2 [8]. Nutlin-binding blocks the interaction with p53, resulting in elevated p53 levels due to reduced turn-over. As several Nutlin-like small molecules are in advanced (pre)clinical development [16-18], it is important to have the means of both correctly anticipating and circumventing possible clinical resistance arising from mutations in HDM2. We have recently described mutations in HDM2 which confer resistance to Nutlin [19]. These mutations render HDM2 able to repress p53 transactivation activity in the presence of otherwise inhibitory Nutlin concentrations. Several of these mutations are in residues either comprising or lying proximal to the N-terminal domain hydrophobic pocket, and simulations propose they function by selectively discriminating against Nutlin binding. We hypothesized that these mutations could be overcome through iterative structure-guided chemical modification of Nutlin, or the use of antagonists with a larger interaction footprint. 

Stapled peptides are a relatively new class of macrocyclic compounds with promising drug-like properties [20]. The introduction of a covalent linkage bridging adjacent turns of an alpha helical peptide (the “staple”), can pre-stabilize the conformer(s) preferentially adopted when it binds a target protein. Stapling increases affinity by reducing the entropic cost of binding, imparts proteolytic stability / increased in vivo half-life, and in certain cases permits adjunct-free cellular uptake [21-23]. Stapled peptide analogues of Nutlin that target the N-terminal domain of HDM2 have been described [9,24], and these mimic the contiguous stretch of p53 (residues 18 to 26) that bind the N-terminal hydrophobic pocket in an α-helical conformation [25-27]. As these stapled peptides form significantly increased contacts with HDM2 compared to Nutlin [28,29], they may prove recalcitrant to mutations that reduce Nutlin efficacy. 

Our data indicates this to be the case, as shown both experimentally and further rationalized by molecular dynamics simulations. The ability of stapled peptides to form comparatively more contacts with target proteins may therefore prove detrimental to the emergence of acquired resistance should this drug-class enter the clinic.

## Materials and Methods

Unless otherwise specified, all oligonucleotides used in this work were from 1^st^ Base (Singapore), restriction enzymes from NEB and chemical reagents from Sigma. Nutlin-3A was from Calbiochem. The stapled peptides PM2, PM2CON and MO11 (>90% purity) were from AnaSpec (USA). 

### Primers

1) HDM2-P20L-QC1: 5'-CCACCTCACAGATTCTAGCTTCGGAACAAGA -3'

2) HDM2-P20L-QC2: 5'-TCTTGTTCCGAAGCTAGAATCTGTGAGGTGG -3'

3) HDM2-Q24R-QC1: 5’-TTCCAGCTTCGGAACGAGAGACCCTGGTTAG -3’

4) HDM2-Q24R-QC2: 5’-CTAACCAGGGTCTCTCGTTCCGAAGCTGGAA -3’

5) HDM2-M62A-1: 5’-CTTGGCCAGTATATTGCGACTAAACGATTATATG-3’

6) HDM2-M62A-2: 5’-CATATAATCGTTTAGTCGCAATATACTGGCCAAG-3’

7) petF2: 5′-CATCGGTGATGTCGGCGAT-3′

8) petR: 5′-GATATAGTTCCTCCTTTCAGCA-3′

9) h_p21_Forward: 5’-GAGGCCGGGATGAGTTGGGAGGAG -3’

10) h_p21_Reverse: 5’-CAGCCGGCGTTTGGAGTGGTAGAA -3’

11) h_p53_forward: 5’-CCCCTCCTGGCCCCTGTCATCTTC -3’

12) h_p53_Reverse: 5’-GCAGCGCCTCACAACCTCCGTCAT -3’

13) h_b-actin_forward: 5’-TCACCCACACTGTGCCCATCTACGA -3’

14) h_b-actin_reverse: 5’-CAGCGGAACCGCTCATTGCCAATGG -3’

15) h_Gadd45alpha_forward: 5’-GAGAGCAGAAGACCGAAAGGA -3’

16) h_Gadd45alpha_reverse: 5’-CAGTGATCGTGCGCTGACT -3’

17) h_14-3-3sigma_forward: 5’-ACTACGAGATCGCCAACAGC -3’

18) h-14-3-3sigma_reverse: 5’-CAGTGTCAGGTTGTCTCGCA -3’

### Vector construction

Single mutant HDM2 clones were generated by Quickchange mutagenesis (Stratagene) of parental HDM2-PET22b using appropriate primers 1-6. The constructs were amplified with primers petF2 and petR to make HDM2 amplicons with T7 promoter and ribosome binding site required for *in vitro* transcription-translation (IVT) of wild-type or mutant HDM2. Primers 1-6 were used to introduce mutations into the parental pCMV-HDM2 mammalian expression construct by Quickchange mutagenesis. Both the HDM2-PET22b and pCMV-HDM2 constructs additionally encode a C-terminal HA tag. The plasmid p53-PET22b was also amplified with petF2 and petR to make template for IVT of wild-type p53.

### Immunoprecipitation and Western blot analysis

Protein G beads (Invitrogen) were incubated with anti-HA (1 µg per 10 µL beads) for 1 hour in PBST-3%BSA and subsequently washed twice in PBST-0.1%BSA. IVT-expressed wild-type or mutant HDM2 was incubated with the beads on a rotator for 30 mins. Nutlin or stapled peptides were added at required concentrations and incubation carried out for 30 mins. IVT-expressed p53 was added to the mixture and incubation allowed for 1 hour. Beads were finally washed thrice in PBST-0.1%BSA and thrice with PBS, and bound proteins eluted by resuspension in 20 µL SDS-PAGE loading buffer and incubation at 95°C for 5 minutes. Both the eluates and inputs were subjected to electrophoresis, transferred to nitrocellulose membranes and probed for p53 with horseradish peroxidise conjugated DO1 antibody (Santa Cruz) or for HDM2 with anti-HA antibody followed by rabbit anti-mouse (Dakocytomation). 

### Cell culture

Mouse embryonic fibroblast p53/Mdm2 double-knockout (DKO) cells (a kind gift from Guillermina Lozano) [30] and H1299 p53^−/−^ cells [31] were maintained in Dulbecco's modified Eagle's medium (DMEM) with 10% (v/v) foetal calf serum (FCS) and 1% (v/v) penicillin/streptomycin. The cells were seeded at 1.0 × 10^5^ cells/well in 6-well plates, 24 hours prior to transfection. Cells were co-transfected with wild-type or mutant HDM2 plasmid, p53-pcDNA plasmid, LacZ reporter plasmid and luciferase plasmid using TurboFect transfection reagent (Thermo Scientific) according to the manufacturer’s instructions. HCT116 p53^+/+^ cells [32] were maintained in McCoy’s 5A medium with 10% (v/v) foetal calf serum (FCS) and 1% (v/v) penicillin/streptomycin. The cells were seeded at 3.5 × 10^5^ cells/well in 6-well plates, 24 hours prior to transfection. Cells were transfected with wild-type or mutant HDM2 plasmid using lipofectamine (Invitrogen) according to the manufacturer's instructions. Nutlin or stapled peptides were added to selected wells at required concentrations 4.5 hours post-transfection. In all cases, the total amount of plasmid DNA transfected per well was equilibrated by addition of the parental vector pcDNA3.1a(+).

### β-Galactosidase assay and Western blot analysis

DKO cells were harvested 24hours after transfection and β-galactosidase activities were assessed using the Dual-light System (Applied Biosystems) according to the manufacturer's protocol. The β-galactosidase activity was normalized with luciferase activity for each sample. To check for expression levels of relevant proteins via Western blot, 5 µg of the cell lysates were probed for p53 with horseradish peroxidise conjugated DO1 antibody, for HDM2 and actin with anti-HA antibody and AC15 antibody respectively followed by rabbit anti-mouse.

### Protein expression and purification

DNA encoding HDM2 (amino acids 1–125) was ligated into the GST fusion expression vector pGEX- 6P-1 (GE Lifesciences) via BamH1 and Nde1 double digest. Mutants of HDM2 (P20L, Q24R and M62A) were made using the QuickChange site-directed mutagenesis kit (Strategene) and appropriate primers 1-6. BL21 DE3 competent bacteria were then transformed with the GST tagged HDM2 (1–125) constructs. Cells harbouring the GST fusion constructs were grown in LB medium at 37°C to an OD600 of ~0.6 and induction was carried out with 1 mM IPTG at room temperature. Cells were harvested by centrifugation and the cell pellets were resuspended in 50 mM Tris pH 8.0, 10% sucrose and then sonicated. The sample was next centrifuged for 60 mins at 17,000 g at 4°C. The supernatant was applied to a 5 mL FF GST column (Amersham) pre-equilibrated in wash buffer (50 mM Tris-HCl pH 8.0, 150 mM NaCl, 1 mM DTT). The column was then further washed by 6 volumes of wash buffer. HDM2 constructs were then purified from the column by cleavage with Precission protease (GE Lifesciences). 10 units of precission protease, in one column volume of wash buffer, were injected onto the column. The cleavage reaction was allowed to proceed overnight at 4°C. The cleaved protein was then eluted off the column with wash buffer. Protein fractions were analyzed with SDS page gel and concentrated using Centricon (3.5 kDa MWCO) concentrator. The protein samples were then dialyzed into buffer A solution (20 mM Bis-Tris, pH 6.5, 1 mM DTT) using HiPrep 26/10 Desalting column, and loaded onto a ResourceS 1 mL column pre-equilibrated in buffer A. The column was then washed in 6 column volumes of buffer A and bound protein was eluted with a linear gradient of 1 M NaCL over 30 column volumes. Protein fractions were analyzed with SDS page gel and concentrated using a Centricon (3.5 kDa MWCO) concentrator, Millipore. The cleaved HDM2 constructs were purified to ~90% purity. Protein concentration was determined using A280 with extinction coefficients of 10430 M-1 cm-1 for the HDM2 (1–125) constructs.

### mRNA quantification

Total RNA was prepared from appropriately treated HCT116 p53^+/+^ cells using the RNeasy Mini Kit (QIAGEN). Reverse transcription was performed using SuperScript™ First-Strand Synthesis System (Invitrogen) with random hexamers. Real-time PCR assays (with appropriate primers 9-18) were carried out using the iQ SYBR Green Supermix (Bio-Rad) on the Bio-Rad CFX384 real-time PCR detection system. Experimental Ct values were normalized to β-actin and relative mRNA expression was calculated versus a reference sample. Data is shown as fold change in gene expression by RT-qPCR (Δ^Δ^Ct method).

### Flourescence anisotropy

Apparent Kds of Nutlin and stapled peptides were determined by fluorescence anisotropy as previously described [24] using purified HDM2 (1-125) and carboxyfluorescein (FAM) labeled 12-1 peptide (FAM-RFMDYWEGL-NH2) [33]. Readings were carried out using the Envision Multilabel Reader (PerkinElmer). All experiments were carried out in PBS (2.7 mM KCl, 137 mM NaCl, 10 mM Na2HPO4 and 2 mM KH2PO4 (pH 7.4)), 3% DMSO and 0.1% Tween 20 buffer. All titrations were carried out in triplicate. Curve-fitting was carried out using Prism 4.0 (GraphPad).

### F2H Co-Localization Assay

Transgenic BHK cells [34] were co-transfected with plasmids encoding the bait p53 (amino acids 1-81) fusion protein and different prey HDM2 (amino acids 7-134) fusion proteins overnight in 96 multiwell plates (µClear Greiner Bio-One, Germany) using the Lipofectamine 2000 (Life Technologies) reverse transfection protocol according to manufacturer’s instructions with 0.2 µg DNA and 0.4 µL Lipofectamine 2000 per well. Cells were incubated with a dilution series of Nutlin or stapled peptides for 1 hour at 37°C, 5% CO_2_. 

Interaction (%) was determined as the ratio of cells showing co-localization of fluorescent signals at the nuclear spot to the total number of evaluated cells. To enable comparison between the experiments, a normalization step was performed. For each independent experiment, the interaction ratios in treated wells were normalized to the interaction ratios in control (untreated) wells:

I % = 100% * Interactions_treated/Interactions_untreated

In this way, the control values in each experiment are taken to be 100%. For automated image acquisition an INCell Analyzer 1000 with a 20X objective (GE Healthcare) was used. Automated image segmentation and analysis was performed with the corresponding INCell Workstation 3.6 software. At least 100 co-transfected cells were analyzed per well. Titrations were carried out independently three to five times.

### In silico simulation studies

The mutations P20L and Q24R lie in the flexible lid region of HDM2 and are missing from the crystal structures of HDM2 (1YCR, 1 RV1) [35,36]. In order to examine the dynamics of the full N-terminal region of HDM2 we grafted 11 conformations of the lid (residues 1-24) from the ensemble of NMR structures (1Z1M)[37] onto 1YCR (residues 25-109). The 11 structures were chosen visually to represent the 3 major states: open, closed and partially open. In addition, there is a recent crystal structure of HDM2 (residues 6-109) that has become available (in complex with a small molecule; PDB code 4HBM, resolved at 1.9Å)[38] with an ordered lid and so we also created a 12^th^ structure of 1YCR where we grafted this lid (only from residues 6-24). The 12 structures generated (wild-type) and the P20L and Q24R mutants generated were all subject to 20 ns molecular dynamics simulations each, in 3 states: apo, complex with p53 peptide and complex with Nutlin, totaling a simulation time of 720 ns for the wild type and for each mutant. A shorter HDM2 was used for HDM2-stapled peptide complexes. For this, residues 19-24 of HDM2 were crafted from 4ERF resolved at 2.0Å [39] on to 1YCR, so as the initial structure includes residues 19 -109 of HDM2. The staple was built using Xleap module in Amber and the parameters were derived from antechamber [40,41] module in Amber. Nutlin parameters were also derived using antechamber. Molecular dynamics simulations were performed with the SANDER module of the AMBER11 [42] package employing the all-atom Cornell force field [43]. Simulations were also carried out for p53, PM2 and M011 peptides bound to HDM2 (19-109) and its mutants (Q24R and M62A). All systems were prepared as described before [44] and simulated for 100 ns at constant temperature (300K) and pressure (1 atm) and structures were stored every 10 ps. The computational alanine-scanning methodology [45] is based on the assumption that replacing the original residue with an alanine will only introduce local changes and not cause a large conformational change to alter the binding mode. Trajectories were sampled every 100 ps for computational alanine scanning using the MM-PBSA post-processing module in amber11. Alanine mutant structures were generated by modifying each residue of the receptor at the C_γ_ atom and by replacing the C_γ_ atom with a hydrogen atom with appropriate distance at the C_γ_-C_β_ bond. PyMOL [46] and Visual Molecular Dynamics [47] (VMD) were used for visualizations. 

All the molecular dynamics (MD) simulations presented were judged to be stable as evidenced by the time dependant evolution of RMSD (Figure S1 in File S1, a-f) and radius of gyration (Figure S1 in File S1, g–l). Conformations extracted from the MD trajectory were subjected to clustering analysis based on RMSD of the lid (1-24) region.

### Statistical analysis

For the F2H assay, significance (t-test) is denoted relative to the percentage interactions observed for wild-type HDM2 under the indicated treatment conditions. For transcript analysis by real-time PCR, two-way ANOVA with Bonferroni post test was performed using GraphPad Prism software. 

## Results

**Figure 1:**
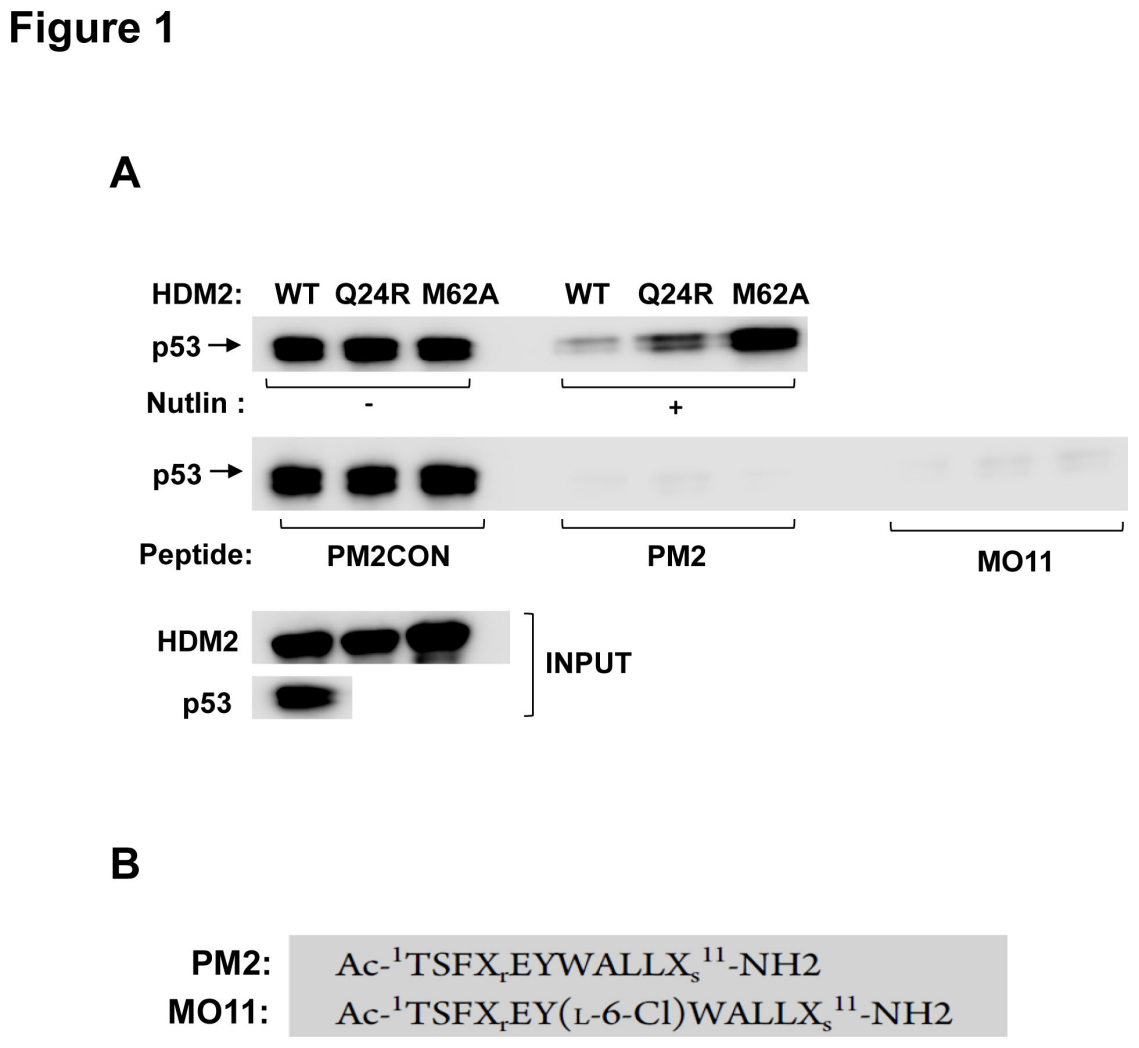
Nutlin-resistant HDM2 variants are inhibited by stapled peptides. A, In vitro pull-down assay shows that Nutlin (10 µM) inhibits p53-HDM2 interaction but is less effective for the Q24R and M62A HDM2 variants (top row). Stapled peptides PM2 and MO11 (10 µM) show inhibition of p53 binding to both WT and mutant HDM2 (second row). 10% of respective HDM2 inputs loaded. PM2CON is negative control stapled peptide. B, Sequences of stapled peptides PM2 and MO11. The residues at positions 3,7,9 of PM2 are mutated to alanine in PM2CON. “X” denotes staple tethering sites. A chlorine atom is added to the C6 position of W7 in MO11.

We first carried out pull-down assays using in vitro expressed proteins to investigate disruption of the HDM2-p53 interaction by Nutlin and the stapled peptides PM2 and MO11 (Figure 1) [24]. These have been designed to target the same hydrophobic cleft of HDM2 to which Nutlin binds. Either wild-type or mutant (M62A and Q24R) HDM2 was captured on beads followed by incubation with either Nutlin or stapled peptide. p53 was subsequently added, and interaction with HDM2 determined by Western blot. The results in Figure 1 indicate strong repression of the HDM2-p53 interaction by both Nutlin and the stapled peptides. As previously described, the M62A and Q24R mutants showed resistance to Nutlin, with increased p53 being pulled down compared to wild-type HDM2 [19]. In striking comparison, the stapled peptides PM2 and MO11 were able to abrogate the mutant HDM2-p53 interaction as efficiently as Nutlin inhibits the wild-type HDM2-p53 interaction. A control stapled peptide PM2CON (PM2 with 3 critical contact residues mutated to alanine) had no effect on binding of p53 to HDM2.

**Figure 2:**
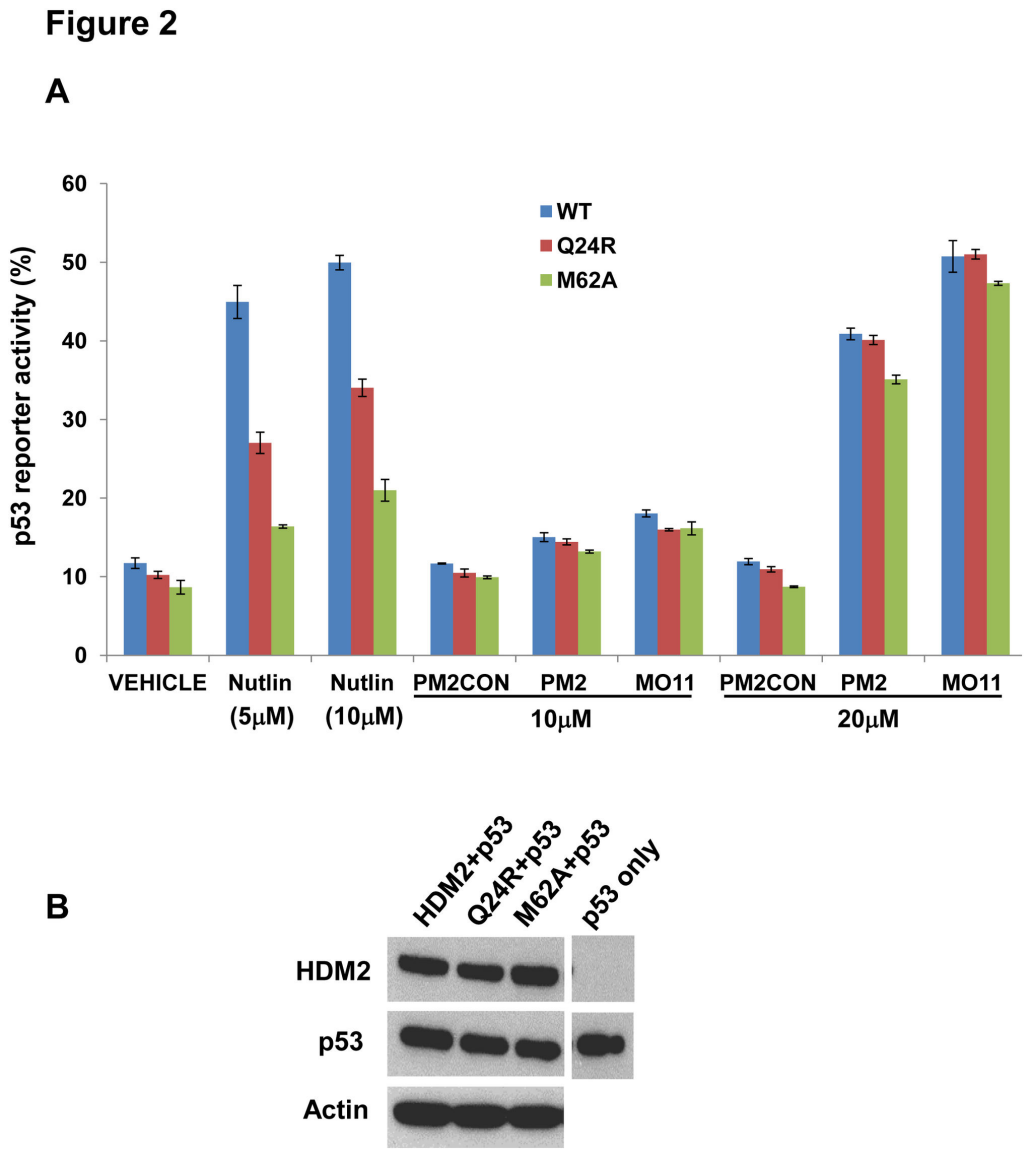
Stapled peptides inhibit wild-type and mutant HDM2 function in p53/MDM2-null DKO cells. A, Wild-type and indicated HDM2 mutants were co-transfected with p53 and p53 reporter gene activity (expressed as percentage of activity observed with p53-only transfection) measured in the presence of Nutlin (0/5/10 µM) and the stapled peptides PM2CON,PM2 and MO11 (10/20 µM). Data represents mean ± SD (n=2). B, Western blots indicating expression levels of HDM2 variants and p53 co-transfected into DKO cells.

Reporter assays in the p53/MDM2-null DKO cell line were carried out to measure p53 transactivation function in the presence of HDM2 and Nutlin/stapled peptides. The results show a clear difference in the ability of Nutlin and the stapled peptides to antagonize mutant HDM2 function (Figure 2). In the absence of antagonist, p53 function was reduced ~ 90% by wild-type and mutant HDM2. Addition of Nutlin (10 µM) restored p53 activity to 50% that seen in absence of HDM2 co-transfection. For the Nutlin-resistant Q24R and M62A mutants, activity was restored to only 34% and 21% respectively. In contrast, the stapled peptides behaved essentially like Nutlin in disruption of the wild-type HDM2-p53 interaction at the higher dose tested (20 µM). PM2 restored activity to 41% whilst the more potent MO11 restored activity to 51%. Notably, the stapled peptides were able to efficiently antagonize the HDM2 mutants. In the case of Q24R, activity was restored to the same level as for inhibition of wild-type HDM2. For M62A, activity was restored to 35% and 47% by PM2 and MO11 respectively. 

**Figure 3:**
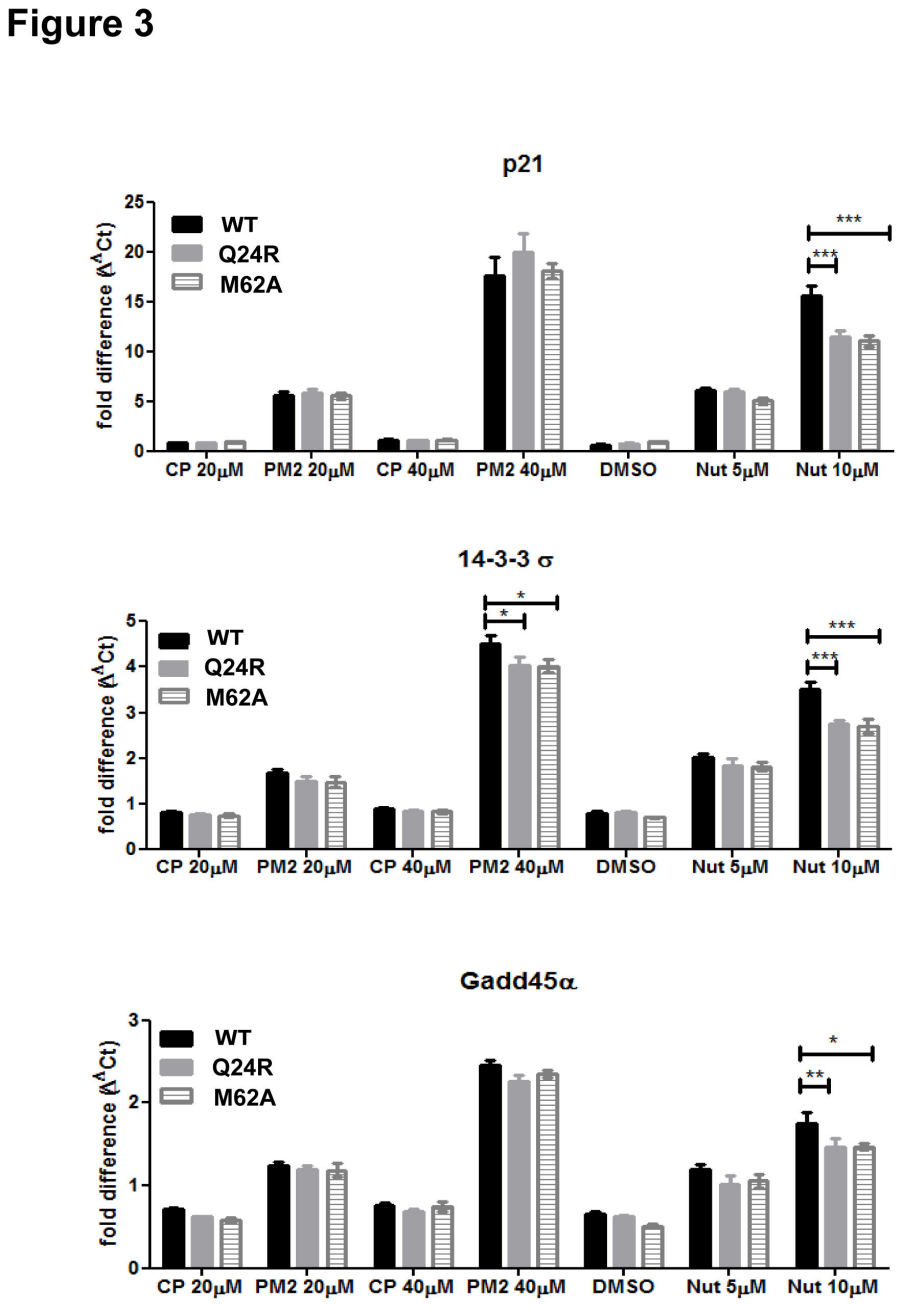
Stapled peptides inhibit wild-type and mutant HDM2 function in HCT116 p53^+/+^ cells as measured by transcriptional response of p53-regulated genes. Concentration-dependent effect of Nutlin (0-10 µM) or DMSO vehicle and stapled peptide PM2 or inactive control peptide (CP) on expression of *p21*, *14-3-3σ*and *gadd45α* genes determined at 24 h post-transfection of HDM2 (WT) and indicated HDM2 mutants. Data shows fold change in gene expression by RT-qPCR (Ct method) c.f. vehicle or control peptide treated cells transfected with pcDNA empty vector. * p < 0.05, ** p < 0.01, *** p < 0.001 (WT versus respective mutant). Data represents mean ± SEM (n=2).

The behavior of the different ligands with respect to regulation of endogenous p53-dependent genes was next investigated in HCT116 p53^+/+^ cells (Figure 3). Wild-type or variant HDM2 was transfected and cells treated with either Nutlin or stapled peptide PM2. p53 activation of *p21*, *gadd45α* and *14-3-3σ* transcript levels [48] [49,50] was measured by qPCR. In the case of Nutlin treatment (10 µM), significant reduction of p53 transcriptional activity was observed for the M62A and Q24R mutants compared to wild-type, consistent with results obtained in DKO cells. The stapled peptide PM2 (40 µM) did not discriminate significantly between inhibition of wild-type and mutant HDM2 with regards to up-regulation of the *p21* and *Gadd45α* genes. In the case of the *14-3-3σ* gene, some resistance to PM2 was observed for the mutants, although this was not as pronounced when compared to Nutlin treatment. No significant differences in expression of the HDM2 mutants was observed compared to wild-type in this cell line (Figure S2 in File S1). 

**Figure 4:**
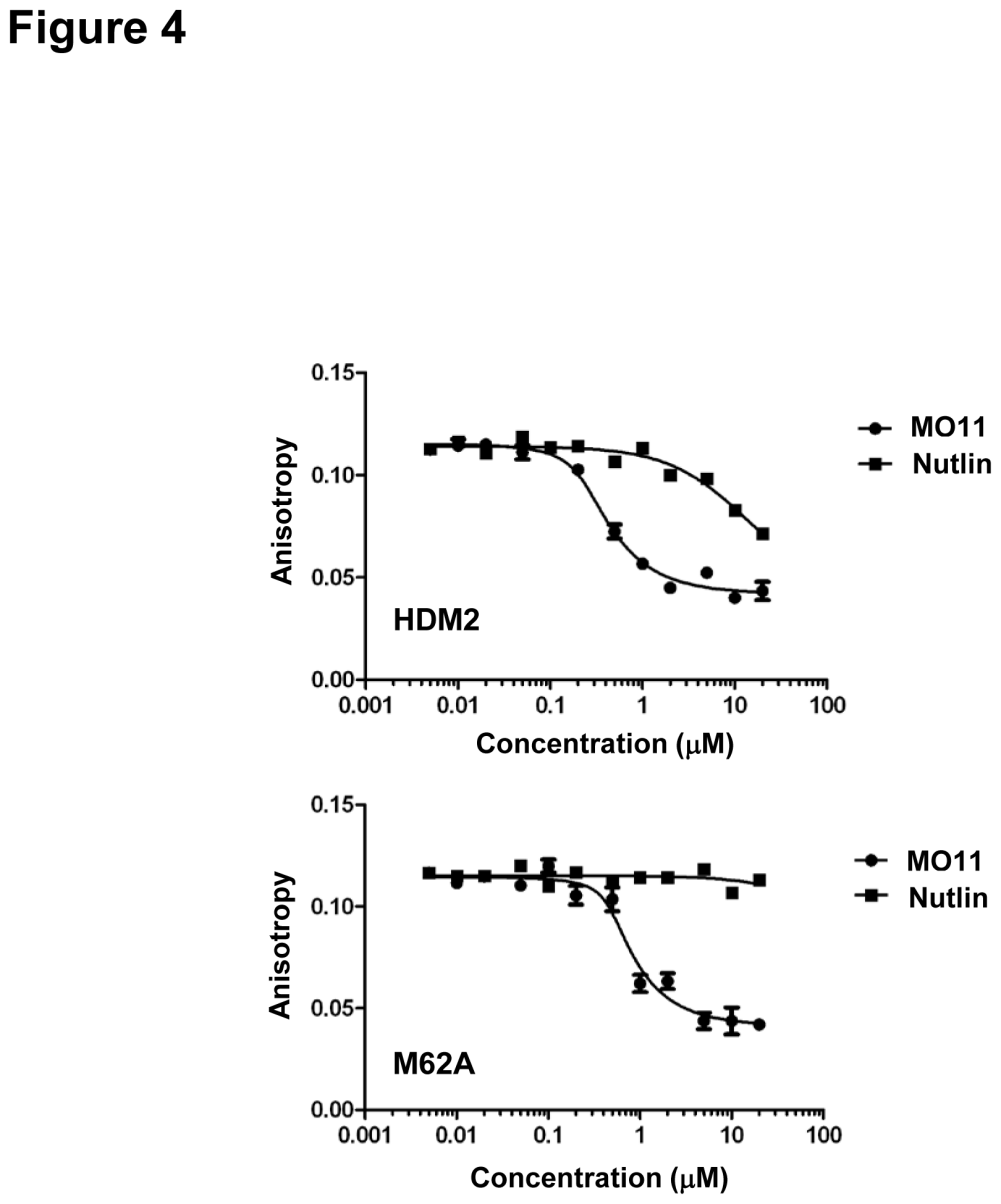
Differential binding of stapled peptide versus Nutlin to HDM2-M62A. Competition titrations of MO11 and Nutlin against FAM-labelled p53-binding peptide 12.1 for binding to wild-type (top) and M62A (bottom) HDM2 mutant. Values represent mean +/- SD (n=2).

Affinity measurements indicated that the M62A mutation significantly reduced affinity for Nutlin compared to wild-type (11426 ± 2490 versus 784.15 ± 11.45 nM respectively)(Figure 4, Table 1). In contrast, very slight perturbation of binding to p53 peptide (29.62 ± 3.03 versus 13.9 ± 4.4 nM for wild-type) and stapled peptide MO11 (18.24 ± 6.14 versus 12.94 ± 3.02 nM for wild-type) was observed. The Q24R and P20L mutants also displayed reduced affinity for Nutlin compared to wild-type (respectively 5282.67 ± 1335.47 and 3041.67 ± 879.71 versus 784.15 ± 11.45 nM). The trend in Nutlin binding affinity for the mutants (M62A < Q24R < P20L) mirrors the resistance phenotypes observed for these mutants in cell-based assays (Figures 2 and 3) [19]. Binding to the stapled peptide MO11 was not perturbed by the Q24R and P20L mutations (respectively 16.94 ± 3.20 and 16.46 ± 4.61 versus 12.94 ± 3.02 nM for wild-type). Similarly, no significant differences were observed for p53 peptide binding to these mutants (10.39 ± 1.30 and 17.22 ± 4.10 versus 13.9 ± 4.4 nM for wild-type). 

**Table 1:** Apparent Kds (nM) of indicated ligands for HDM2 mutants determined by competitive fluorescence anisotropy titrations.

	**P53 peptide**	**MO11**	**Nutlin**
**HDM2**	13.9 ± 4.4	12.94 ± 3.02	784.15 ± 11.45
**M62A**	29.62 ± 3.03	18.24 ± 6.14	11426 ± 2490
**Q24R**	10.39 ± 1.30	16.94 ± 3.20	5282.67 ± 1335.47
**P20L**	17.22 ± 4.10	16.46 ± 4.61	3041.67 ± 879.71

Values represent mean ± SD (n=2-4).

**Figure 5:**
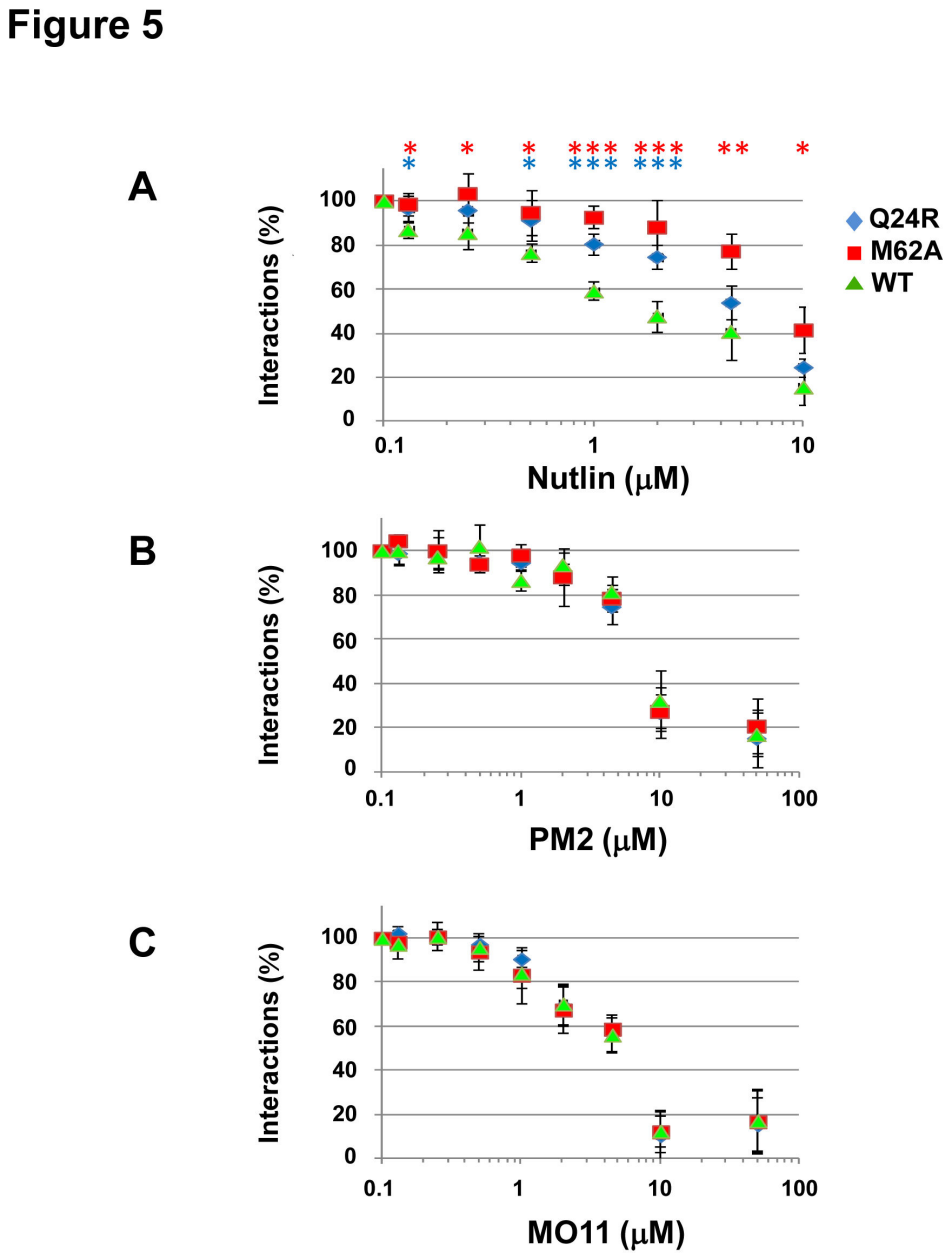
Direct interaction between p53 and HDM2 N-terminal domain inhibited by stapled peptides. F2H assay to investigate the interaction of p53 (bait) with wild-type HDM2 (WT), mutant Q24R and M62A (preys). The F2H assay measures the interaction between two proteins as ratio of cells showing co-localization of bait and prey at the nuclear F2H interaction platform, to cells not showing this co-localization. A. Titration of Nutlin results in increased dissociation of WT-p53 complex compared to Q24R/M62A-p53 complexes. B-C, In contrast, the stapled peptides display equivalent potency on WT and mutant HDM2. Data points represent normalized interaction values expressed as percentage interactions observed in the absence of treatment ± SD (n=4). * p < 0.05, ** p < 0.01, *** p < 0.005 (WT vs M62A, blue asterisks. WT vs Q24R, red asterisks).

The direct cellular binding of the HDM2 wild-type, Q24R and M62A N-terminal domains to p53 was further characterized in the Fluorescent 2-Hybrid (F2H) assay [34]. The F2H assay visualizes the interaction of RFP-tagged HDM2 (amino acids 7-134) with GFP-tagged p53 (amino acids 1-81) at a defined nuclear F2H interaction platform, in specific BHK cells. Dissociation of the complex due to interaction with Nutlin or stapled peptide can be imaged and quantified. Compared to the wild-type HDM2-p53 interaction, addition of Nutlin resulted in reduced dissociation of mutant N-terminal domains from p53, indicating Nutlin resistance (Figure 5). This was particularly evident in the dose range 1-10 µM. In comparison, no significant differences were seen between wild-type and mutant N-terminal domains when the stapled peptides were used to dissociate the complexes. In agreement with the reporter assays (Figure 2), MO11 was more potent than PM2 in disrupting the complex. 

## Discussion

Systematic analysis of small molecule versus peptide binding to target proteins indicates that the former do not take advantage of all the available opportunities for polar contacts, and typically rely on a few anchor points and hydrophobic interactions to achieve high-potency binding [51]. This binding deficit may therefore be readily exploited through point mutation, as seen with the M62A and Q24R mutations in HDM2. In contrast, the peptide-protein binding interface generally employs a more diffuse network of polar interactions, intimating that peptide/peptide-like molecules should be intrinsically more recalcitrant to point mutations in target proteins. Our data supports this notion, as both in vitro and ex vivo assays indicate that point mutants of HDM2 that inhibit Nutlin, but not p53 binding have reduced or no impact on the interaction with stapled peptides. As these mutants were originally selected to retain p53, but not Nutlin binding [19], this shows that the stapled peptides faithfully mimic the endogenous p53 N-terminal domain interaction with HDM2. Further selections are currently under way to determine whether HDM2 resistance to the stapled peptides (but not p53) can be evolved. Based on in silico predictions (see below), it is likely that a higher mutational burden will be required. Furthermore, in the context of the overall p53-HDM2 binding interaction, it is plausible that mutations in the secondary binding interface that selectively increase affinity for p53 (for example V280A [19]) could indirectly confer resistance to stapled peptides. Given the highly allosteric nature of HDM2 [52], mutations in distal domains may also impact on binding of stapled peptides. 

**Figure 6:**
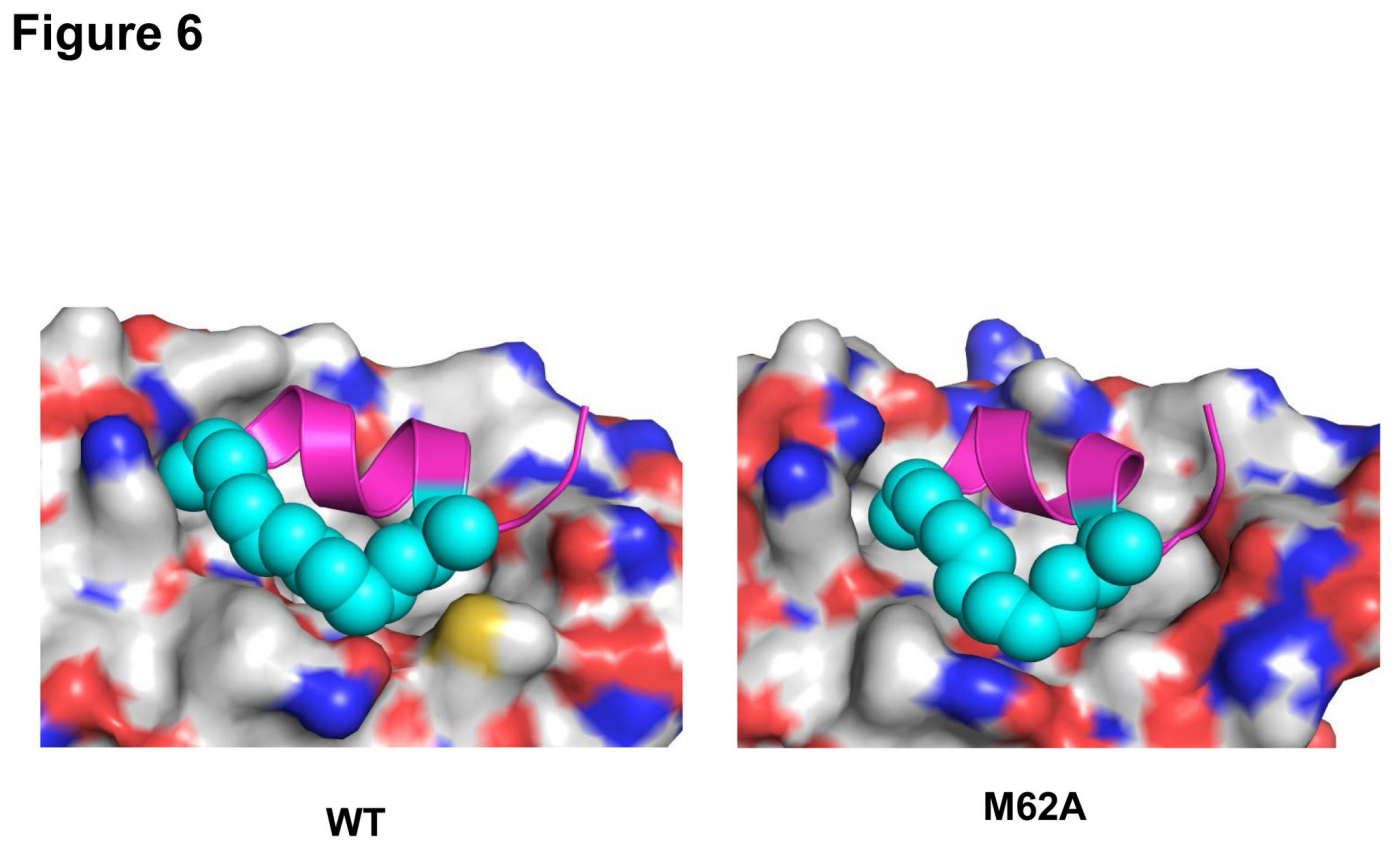
The M62A mutation in HDM2 does not perturb binding of the stapled peptide PM2. Left: Simulations (see Materials and Methods) indicate packing interactions between M62 (yellow) and hydrophobic staple (cyan) of PM2. Right: Mutation to alanine results in loss of these interactions, but numerous other interactions persist between PM2 and hydrophobic cleft of HDM2. The peptidic component of PM2 is depicted in magenta.

Modelling studies show the hydrophobic hydrocarbon chain comprising the staple interacts with HDM2 in the vicinity of M62 (Figure 6). Whilst the M62A mutation impacts negatively on binding of both stapled peptide and p53 compared to wild type HDM2, the presence of additional “fall-back” interactions (apart from M62, see below) results in marginal overall loss of binding by these ligands. Cluster analysis on the stapled peptide bound to HDM2 and the M62A mutant shows that in the majority of the conformations sampled (91%), M62 is in close contact with the staple. Mutation to alanine causes marginal loss of interaction (Figure 6 and Figure S3a in File S1) in the majority (92%) of conformations sampled (Figure S3a in File S1). In the case of Nutlin binding, interaction with M62 contributes significantly to overall binding, and hence major loss of binding occurs when this contact is lost [19]. 

**Figure 7:**
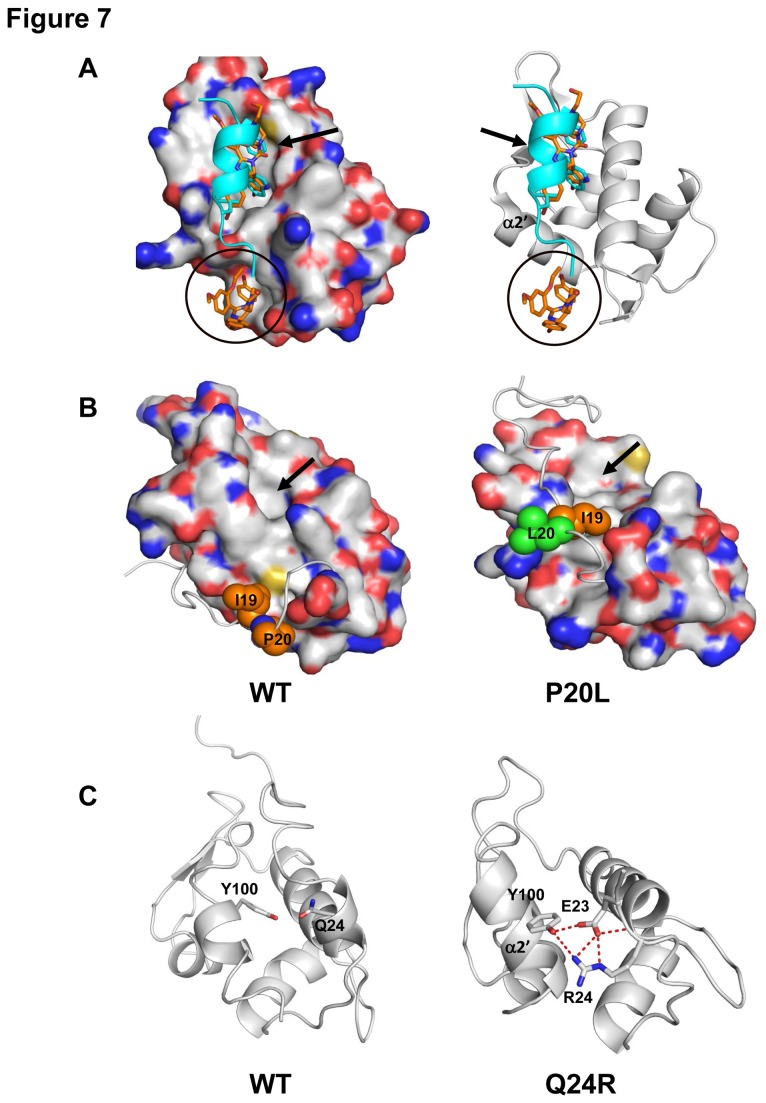
Molecular simulations show negative impact of the P20L and Q24R mutations on the docking of Nutlin to the HDM2 N-terminal domain. A, Space-filling (left) and ribbon (right) depictions of Nutlin (orange) binding the main (arrowed) and secondary (circled) binding sites of HDM2. The p53-peptide (cyan) binding to the main site is overlaid. B, Space-filling model of the HDM2 N-terminal domain showing migration of the lid region when P20 (left, orange sphere,) is mutated to leucine (right, green sphere). Arrow depicts main Nutlin binding site. C, Mutation of Q24 (left) to arginine (right) results in extensive hydrogen bond network with E23 and Y100 and likely occlusion of the secondary Nutlin binding site.

To date, most understanding of the interactions of Nutlin with HDM2 have focused on the “main” site (Figure 7A), and indeed this has been instrumental in the design of small molecules that are now in clinical trials. However, the crystal structure of Nutlin complexed with HDM2 also shows a second molecule of Nutlin that interacts with the α2' region of HDM2 and we call this the “secondary” site (Figure 7A). This interaction has never been deemed important as it was thought to result from crystal contacts. Recently, hints that there may be more to this appeared when Brownian Dynamics simulations demonstrated that in solution, Nutlin would bind to this site also [53]. More recently, H-D exchange data combined with molecular simulations and rationally designed mutagenesis studies have demonstrated that this region near α2', is the site where Nutlin appears to first bind and then shuttle to the "main" binding site [54]. Simulations of the P20L mutation shows that L20 together with I19 packs against the ridge of the p53 binding pocket leading to a partial occlusion of the main binding site (Figure 7B), notably the region where L26 of p53 embeds. Cluster analysis on the apo P20L MD data shows that 84 % of the conformations sampled place the I19-L20 in this position within HDM2 (Figure S3b in File S1; Movie S1 and Movie S2). The distribution plot for the distances between the side chains of P20 - H96, L20 - H96, P20 - Leu54 and L20 - Leu54 shows that L20 is closer to H96 and Leu54 (based on the major population shift in the distances, when compared with P20), which are located close to the ridge of the main and secondary pockets (Figure S4a in File S1). This places a barrier for Nutlin migration from the secondary to the main site and may account for the resistance of this mutant to Nutlin binding. Binding to p53 is retained as the lid only occludes the L26 site; we have previously shown that p53 likely binds with F19 docking first and enabling a crack to propagate [55]. This suggests that in these mutants, p53 and stapled peptides can dock into the open F19 docking site and then slowly edge the lid out. Further simulations are in progress to investigate this.

The Y104G mutation in the secondary Nutlin binding site is recalcitrant to Nutlin binding (yet retains p53 binding), thus suggesting that this binding site indeed may be crucial for Nutlin interactions as hypothesized [54]. Earlier Brownian dynamics simulations have also provided hints that residues in this region (E25 and K51) appear to play key roles in channeling Nutlin into HDM2 [56]. These residues (E25, K51 and Y104) are in closer proximity to the second Nutlin binding site than the primary p53/Nutlin binding site. Studies have shown that K51 of HDM2 interacts with E23 and E25 of HDM2 [57]. Simulations indicate that the Q24R mutation leads to the development of a cationic potential in the region of R24 and will therefore undoubtedly influence the dynamics of E25. In the simulations of Q24R, it is clear that it engages in an extensive hydrogen bond network with E23 and Y100, all in the vicinity of the secondary site (Figure 7C; Figure S3b in File S1; Movie S3 and Movie S4). The distribution plot for the distances between the side chains of Q24 - E23, R24 - E23, Q24 - Y100 and R24 - Y100 shows that the hydrogen bond network with R24 lasts longer (Figure S4b in File S1) than the similar hydrogen bond network with Q24. This has two effects that will likely deter Nutlin “landing”: destabilizing the E25 - K51 salt bridge which perturbs the secondary site and interactions of R24 with E23 and Y100 which occludes the secondary site; the conformations that are sampled account for ~42% of the total sample. The relationship between the kinetics of approach to the secondary site, the migration to the primary site and the thermodynamics of binding (largely determined by residence times or k_off_) at the primary site are not clear yet. The predictive value of conventional methods of drug design that rely only on the free energy of binding at the primary site will undoubtedly be augmented by incorporating kinetic parameters; we are currently exploring this through extensive simulations.

The presented hypothesis derived from the simulation results needs be further tested experimentally by exploring various mutations in the secondary binding site that impact on Nutlin interaction. Furthermore, the binding characteristics of small molecules bulkier than Nutlin [58,59] that might not be able to fit into the second-site should be assessed, in addition to the impact of secondary site lid mutations on binding of these and other compounds [39,60] targeting the HDM2 hydrophobic cleft. 

**Figure 8:**
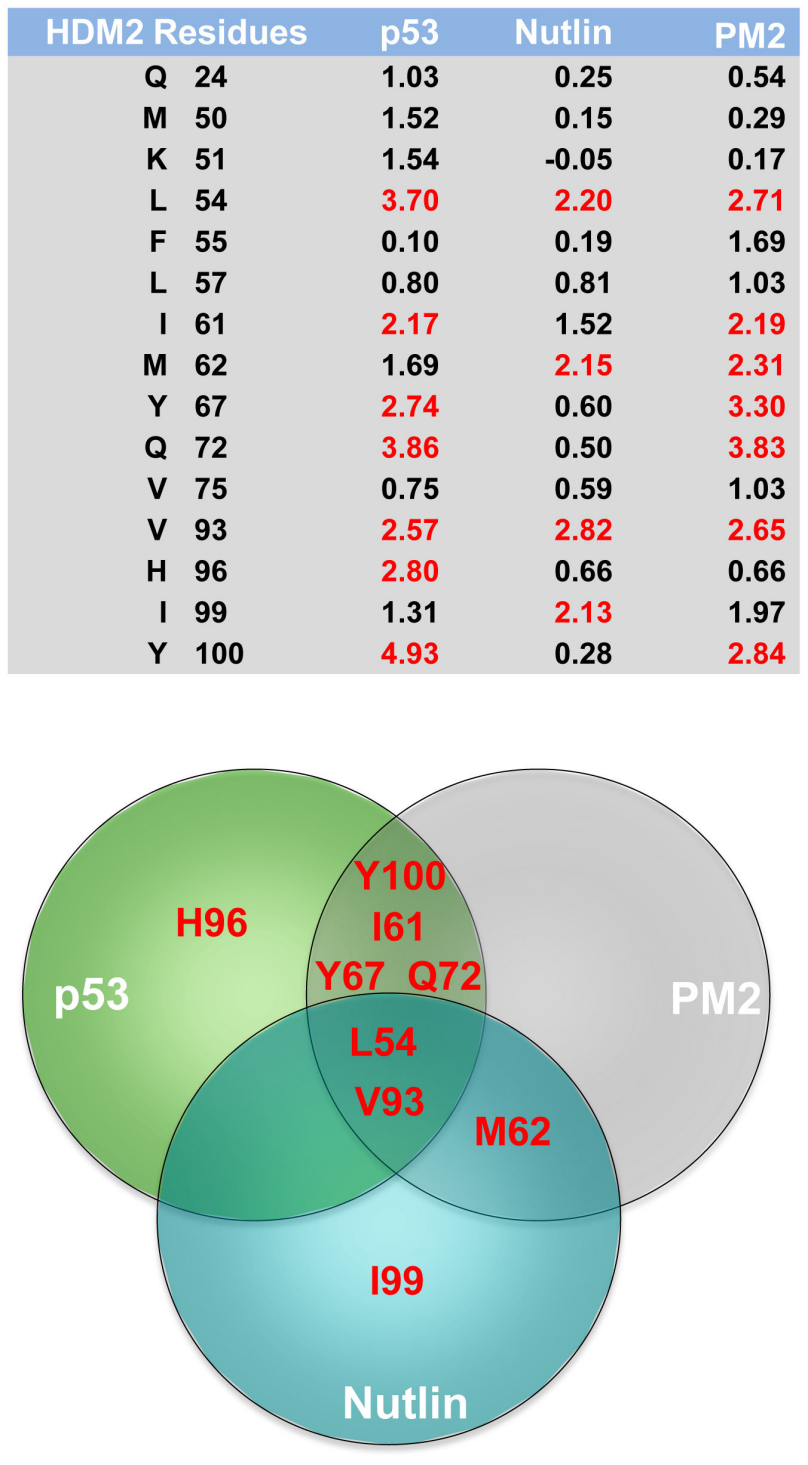
Energetic contribution to the differences in the binding free energies (ΔΔG_Binding_) between wild type HDM2 and alanine mutant variants for binding to the indicated ligands. Residues contributing > 2 kcal/mol are highlighted in red, and are further depicted in Venn diagram below.

Many computational studies have been used to address resistance mutations and their structural and energetic coupling to inhibitors in EGFR kinase [61] and HIV [62,63]. However it would be useful if a method could predict the emergence of mutations at key sites in proteins. Towards this, we begin by asking a simple question: what mutation would enable HDM2 to destabilize interactions with stapled peptide and strengthen them with p53? A computational alanine scan was therefore carried out whereby all the residues in the HDM2 N-terminal domain (except glycine, alanine and proline) were individually mutated to alanine. The effects of point mutations on the interactions with p53 peptide, Nutlin, and stapled peptide were recalculated for the absolute binding free energy for the mutated system. The computational alanine scanning results for selected residues are summarised in Figure 8 and Figure S5 in File S1. Positive and negative values indicate unfavourable and favourable contributions, respectively. Seven residues contribute significantly (> 2 kcal/mol) to binding of p53 peptide and PM2, of which 6 are common to both ligands (Figure 8). The multiplicity of shared anchor points indicates that point mutations selectively discriminating against stapled peptide, but not p53 binding are less probable. In contrast, only four residues (L54, M62, V93, I99) contribute significantly to Nutlin binding. Of these, L54 and V93 are important for binding of all ligands, whilst M62 plays a significant role for PM2 and Nutlin binding. Hence, as shown experimentally for M62, mutation of any of the residues important for Nutlin binding is likely to selectively perturb Nutlin but not p53 binding to HDM2. Whilst M62 is also involved in binding of stapled peptide (Figure 6), the presence of numerous other contact points results in no significant detriment to binding when this amino acid is mutated. It is important to note that this analysis does not account for whether the mutations destabilise the HDM2 fold. However, such mutations would most likely impact negatively on p53 binding, and are thus unlikely to arise. Computational predictions therefore suggest that selective resistance to stapled peptides is unlikely to occur through point mutation in the N-terminal p53-binding pocket of HDM2. However, it is important to further query this hypothesis using both rational and directed evolution approaches.

In this study, we have demonstrated that stapled p53-peptide analogues can function in cells as next-generation ligands capable of reverting a drug-resistant phenotype due to mutation in HDM2. As small molecule HDM2 inhibitors have yet to be approved for clinical use, it remains to be seen whether the resistance mutations identified will manifest. Furthermore, one cannot discount acquired resistance to stapled-peptide analogues should these prove viable therapeutic reagents. In this case, application of both guided and combinatorial selection methods will expedite the development of second-line antagonists.

## Supporting Information

File S1 **This file contains Figure S1-Figure S5.** Figure S1, Root mean squared deviation for apo HDM2 A) wild type, B) P20L, C) Q24R, D) M62A and for HDM2 bound to stapled peptide E) wild type, F) M62A; Radius of gyration for apo HDM2 G) wild type, H) P20L, I)Q24R, J) M62A and for HDM2 bound to stapled peptide K) wild type, L) M62A. Figure S2, Expression levels of HA-tagged wild-type (WT) and indicated HDM2 mutants transfected into HCT116 p53^+/+^ cells and treated with either Nutlin or stapled peptides PM2CON and PM2 as indicated. Figure **S3**, The percentage distribution of the clusters based on the lid (residues 1 to 24) position a) stapled peptide bound to wt-HDM2 and HDM2 (M62A). b) Apo wt-HDM2, HDM2 (P20L) and HDM2 (Q24R). Figure **S4**, The distribution of distances among side chains of P20/L20 with H95,L54 and Q24/R24 with E25,Y100. A) The distances between C_β_ atoms of residues P20 - H96, L20 - H96, P20 - L54, and L20 - L54. B) The distances between C_β_ atoms of residues Q24 - E25, R24 - E25, Q24 - Y100, and R24 - Y100. Figure **S5**, Energetic contribution (kcal/mol) of indicated HDM2 residues to binding of p53 peptide, Nutlin, and stapled peptide PM2 as determined by computational alanine scanning (see Materials and Methods).
(PDF)

Movie S1 **Movie of MD simulation trajectory of Apo wt-HDM2 (residues 25 to 110 are shown in surface; the lid region is shown in cartoon and the residues I19 and P20 are shown in spheres).**
(WMV)

Movie S2 **Movie of MD simulation trajectory of Apo HDM2-P20L (residues 25 to 110 are shown in surface; the lid region is shown in cartoon and the residues I19 and L20 are shown in spheres).**
(WMV)

Movie S3 **Movie of MD simulation trajectory of Apo wt-HDM2 (residues E23, Q24, Y100 and Y104 are shown sticks).**
(WMV)

Movie S4 **Movie of MD simulation trajectory of Apo HDM2-Q24R (residues E23, R24, Y100 and Y104 are shown sticks).**
(WMV)
