# Supplementary figures and images for: Inhibition of Nutlin-Resistant HDM2 Mutants by Stapled Peptides
Source: PLoS One. 2013 Nov 20;8(11):e81068. doi: 10.1371/journal.pone.0081068 (PMC3835680; doi:10.1371/journal.pone.0081068)

Figure S1

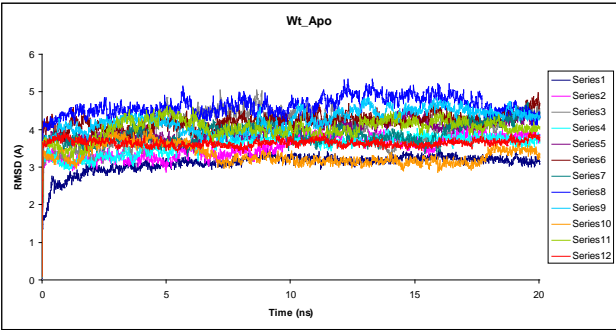

(a)

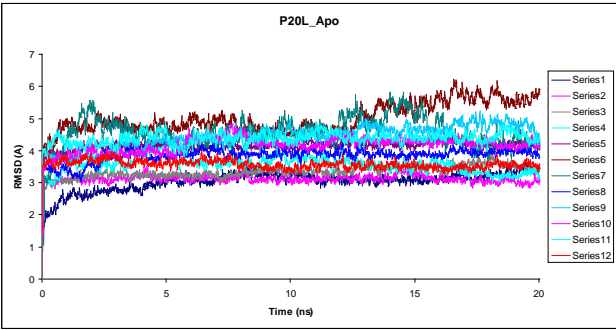

(b)

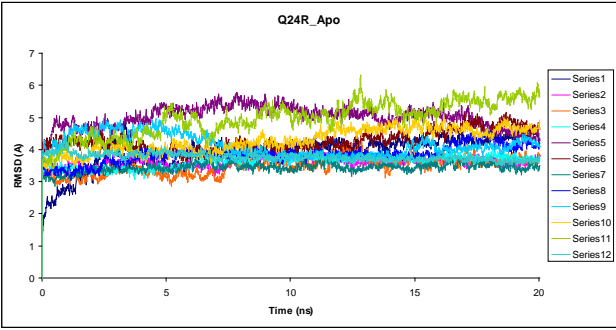

(c)

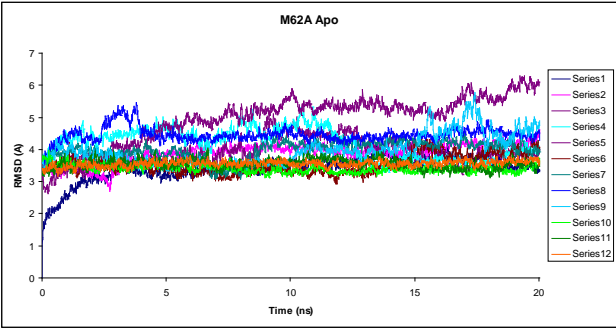

(d)

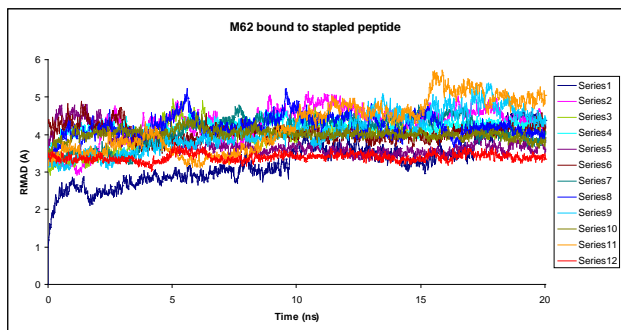

(e)

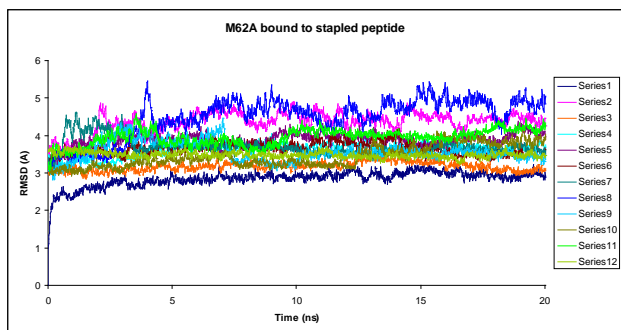

(f)

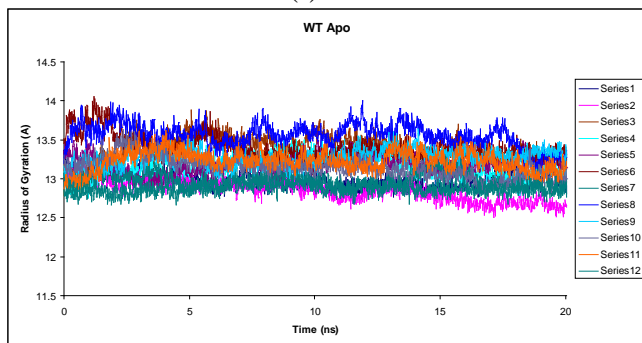

(g)

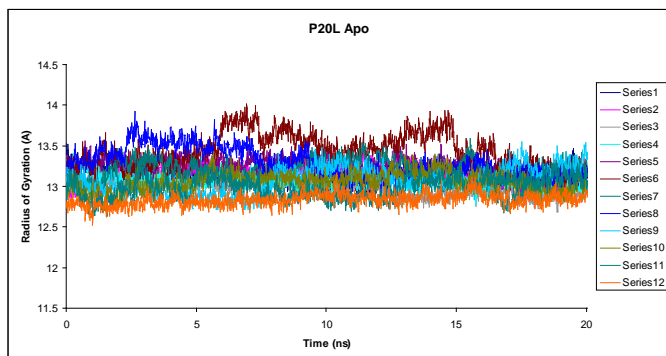

(h)

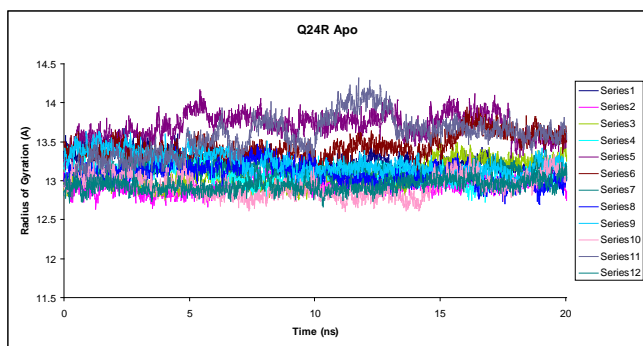

(i)

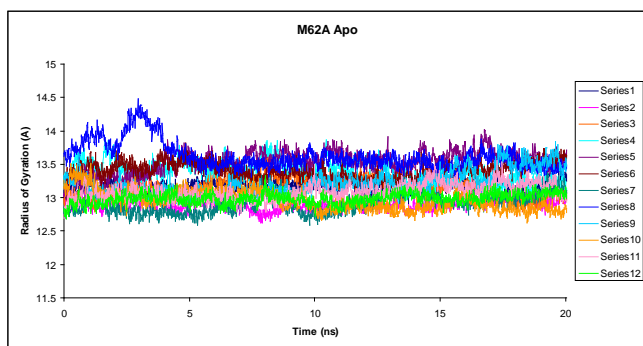

(j)

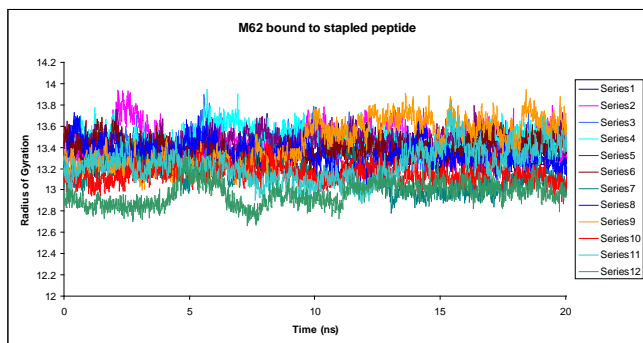

(k)

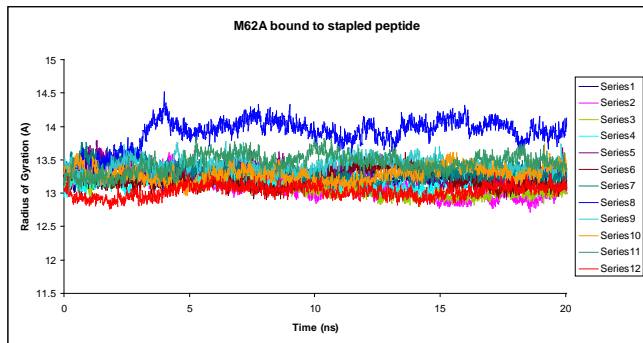

(l)

Figure S2

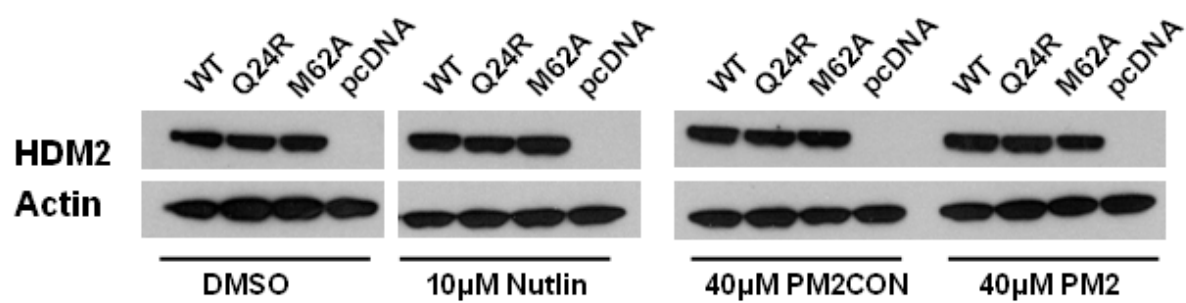

Figure S3a

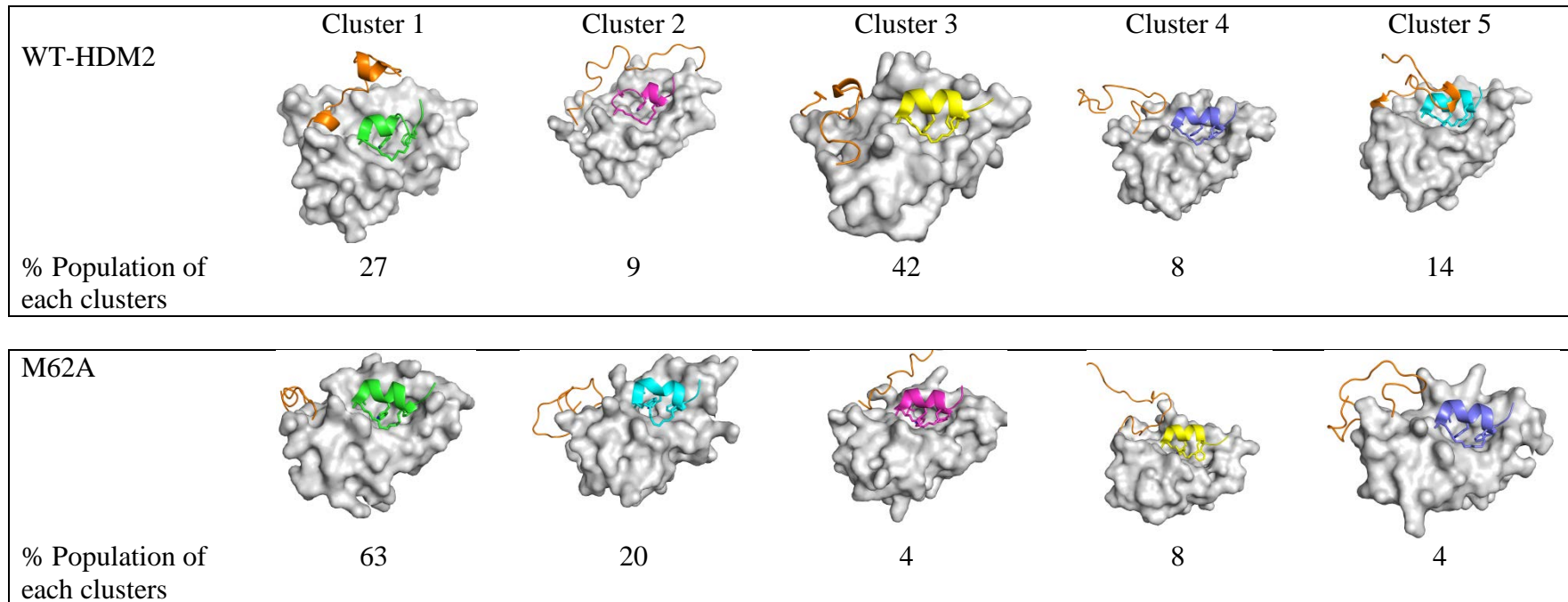

Figure S3b

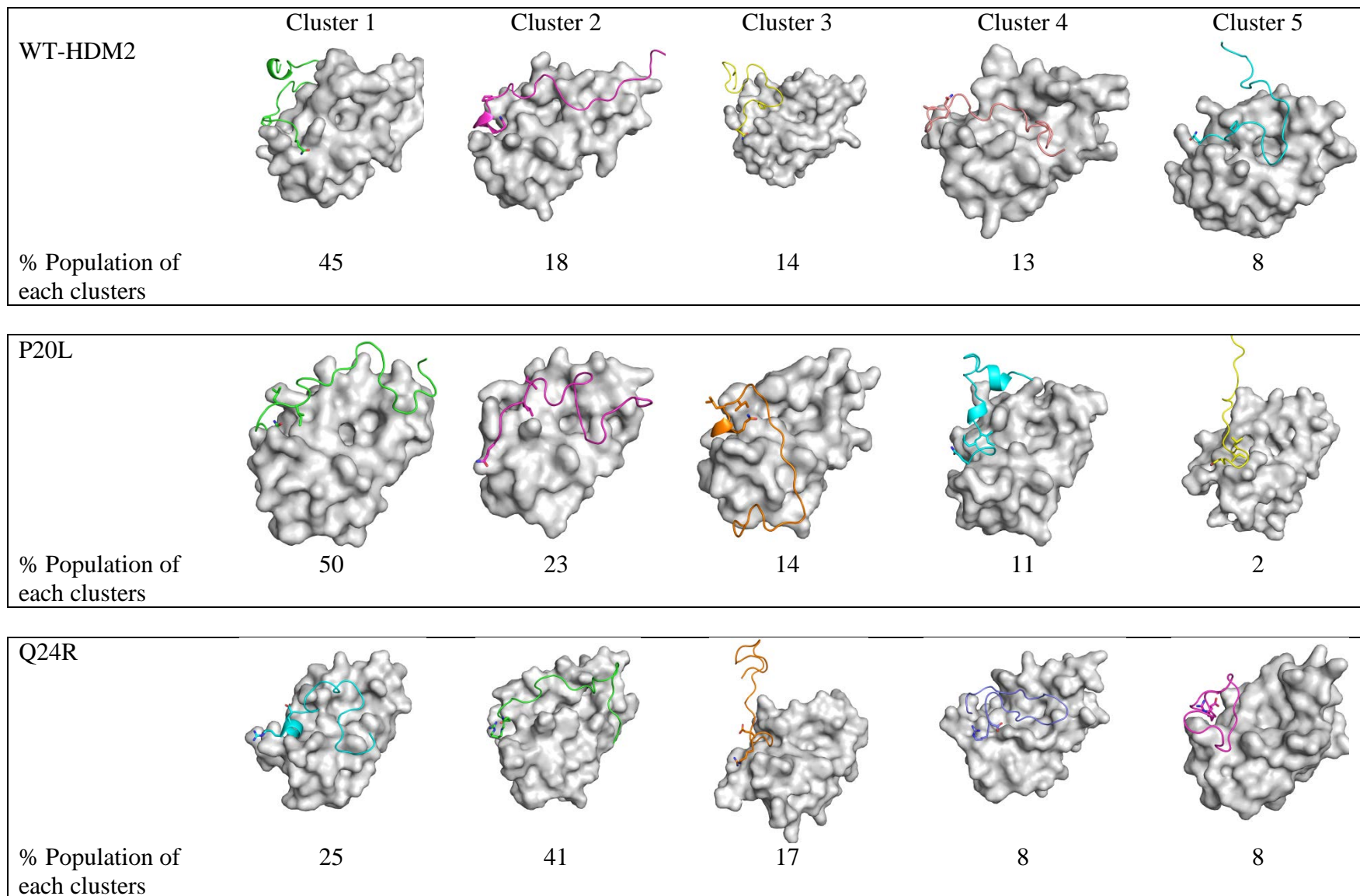

Figure S4a

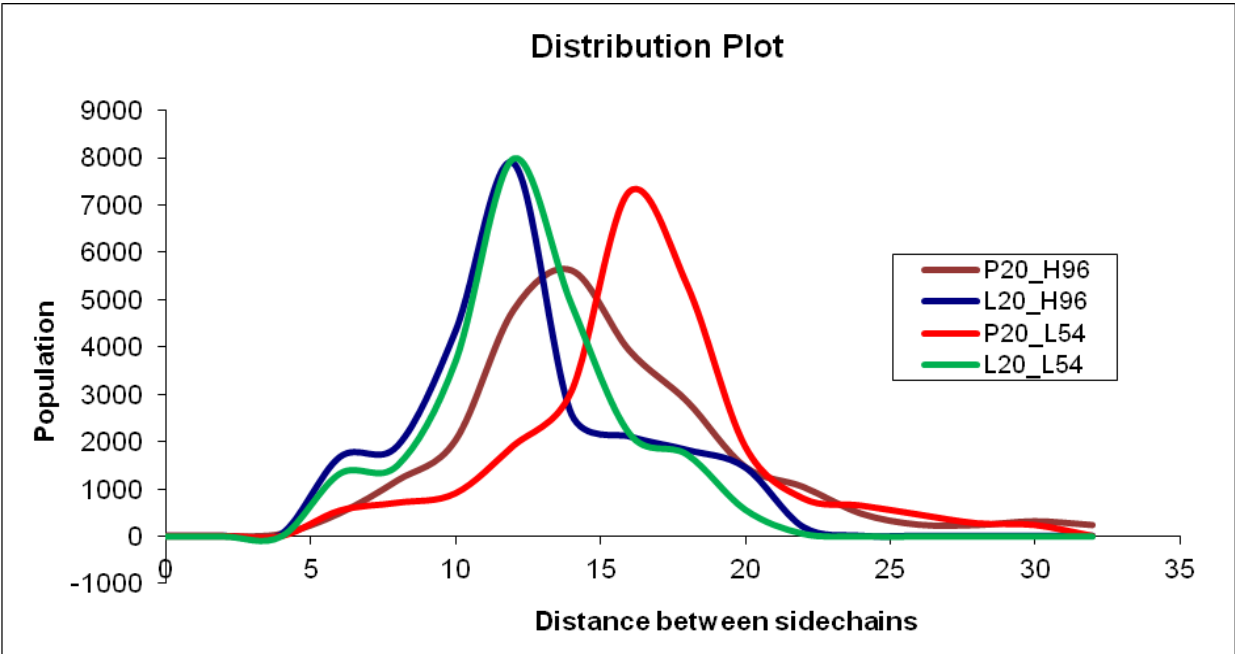

Figure S4b

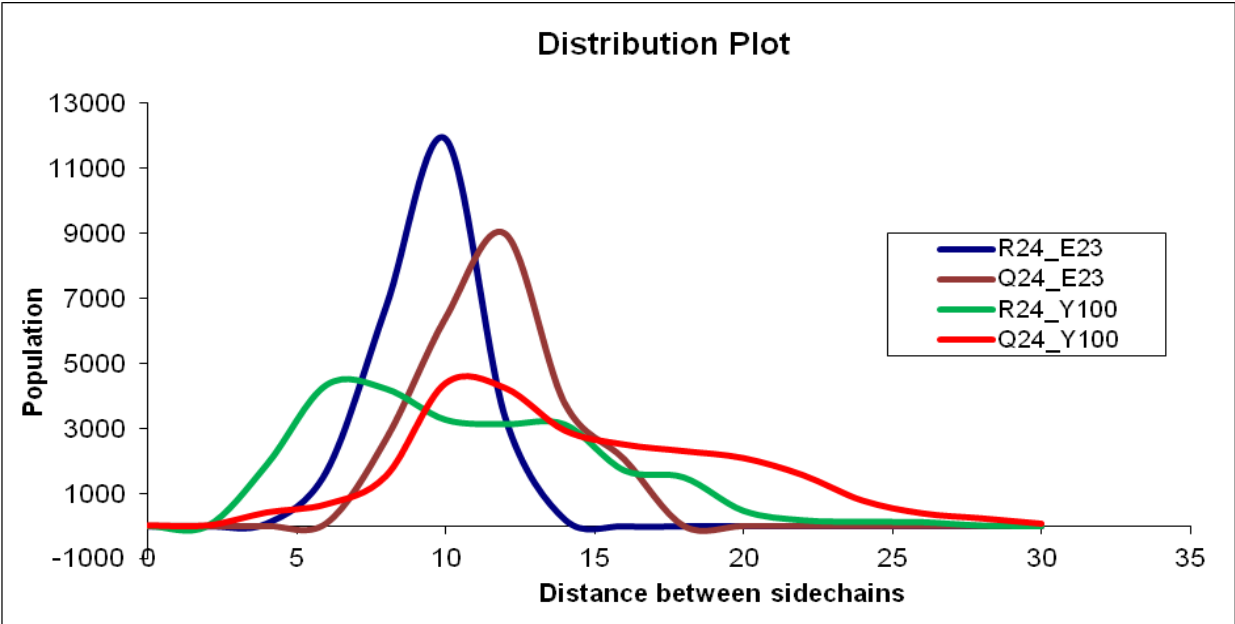

Figure S5

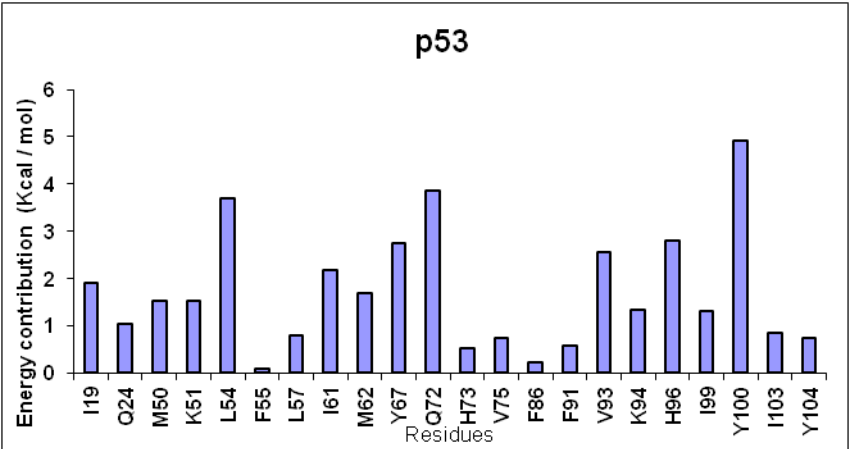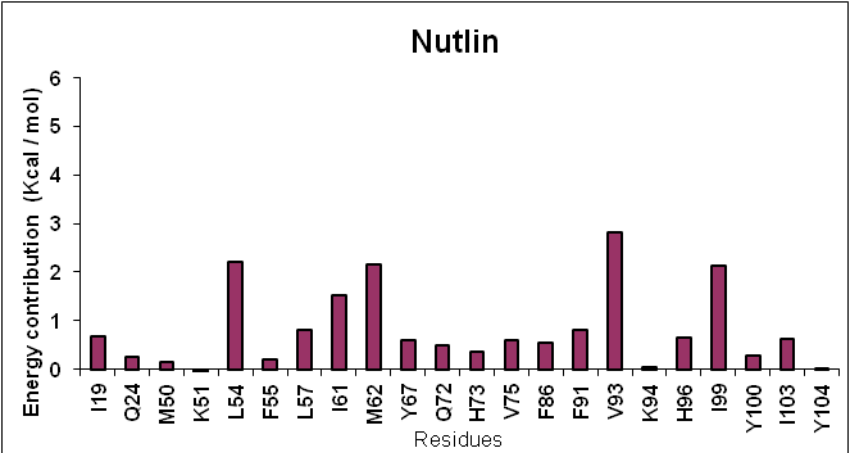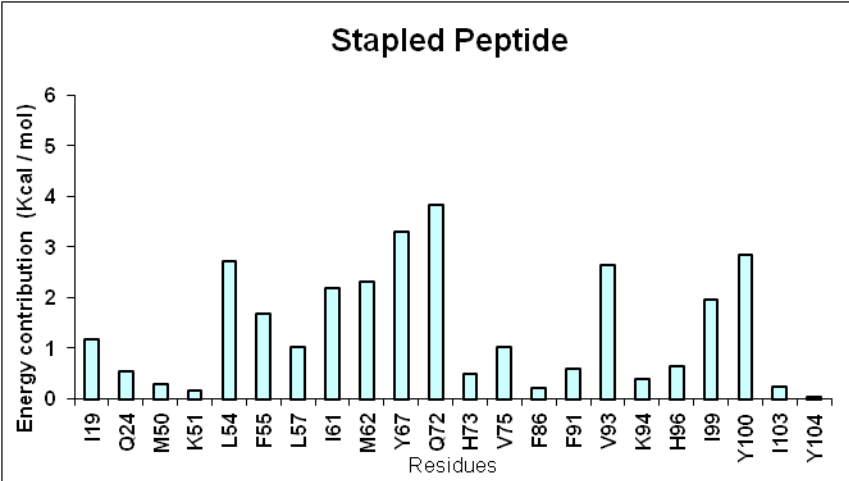

Supplement: File S1 — This file contains Figure S1-Figure S5. Figure S1, Root mean squared deviation for apo HDM2 A) wild type, B) P20L, C) Q24R, D) M62A and for HDM2 bound to stapled peptide E) wild type, F) M62A; Radius of gyration for apo HDM2 G) wild type, H) P20L, I)Q24R, J) M62A and for HDM2 bound to stapled peptide K) wild type, L) M62A. Figure S2, Expression levels of HA-tagged wild-type (WT) and indicated HDM2 mutants transfected into HCT116 p53+/+ cells and treated with either Nutlin or stapled peptides PM2CON and PM2 as indicated. Figure S3, The percentage distribution of the clusters based on the lid (residues 1 to 24) position a) stapled peptide bound to wt-HDM2 and HDM2 (M62A). b) Apo wt-HDM2, HDM2 (P20L) and HDM2 (Q24R). Figure S4, The distribution of distances among side chains of P20/L20 with H95,L54 and Q24/R24 with E25,Y100. A) The distances between Cβ atoms of residues P20 - H96, L20 - H96, P20 - L54, and L20 - L54. B) The distances between Cβ atoms of residues Q24 - E25, R24 - E25, Q24 - Y100, and R24 - Y100. Figure S5, Energetic contribution (kcal/mol) of indicated HDM2 residues to binding of p53 peptide, Nutlin, and stapled peptide PM2 as determined by computational alanine scanning (see Materials and Methods). (PDF) [file pone.0081068.s001.pdf]
